# Enhancing emotional intelligence in Egyptian medical students: a quasi-experimental study using Kirkpatrick’s model

**DOI:** 10.1186/s12909-026-09408-z

**Published:** 2026-05-19

**Authors:** Rania Hadhoud, Omayma Hamed, Maram Hassan

**Affiliations:** 1 Department of Forensic Medicine and Clinical Toxicology, Ain Shams University, Cairo, Egypt; 2 Department of Medical Education, Armed Forces College of Medicine, Cairo, Egypt; 3 Department of Medical Education, Alexandria University, Alexandria, Egypt

**Keywords:** Emotional intelligence, Medical education, Egypt, Kirkpatrick model, Student satisfaction, Quasi-experimental study, Reflective learning, Constructivist pedagogy, SSEIT

## Abstract

**Background:**

Emotional intelligence (EI) is a vital competency in the medical field, contributing to communication, empathy, and resilience. In the Middle East, traditional medical curricula often fail to incorporate EI training, despite its importance being adequately recognized. This study evaluates the impact of a structured elective on emotional intelligence among third-year medical students at an Egyptian medical school.

**Methods:**

A quasi-experimental pre-post design was employed, focusing on the first two levels of the Kirkpatrick evaluation model: Reaction (learner satisfaction) and Learning (acquisition of knowledge and skills). The intervention employed interactive techniques, including small-group discussions, role-playing, reflective writing, and multimedia presentations. Learner satisfaction was assessed using a validated Students’ Evaluation of Teaching (SETs) survey. EI knowledge and competencies were measured with a 22-item knowledge test and the Schutte Self-Report Emotional Intelligence Test (SSEIT) administered before and after the course. Quantitative data were analyzed using paired t-tests, Cohen’s d, and an Agreement Index; qualitative feedback from open-ended questions was analyzed using thematic analysis.

**Results:**

Following the intervention, there were notable improvements in EI knowledge (mean score increased from 11.81 to 14.42, *p* < 0.001) and overall, EI scores (122.36 to 126.69, *p* < 0.001), with significant effect sizes (Cohen’s d = 0.94 and 2.03, respectively). Students expressed high satisfaction with interactive activities and reflective exercises (Agreement Index = 4.9/5). Although some suggested a more integrated approach to clinical scenarios, qualitative feedback highlighted improvements in self-awareness, empathy, and clinical relevance.

**Conclusions:**

A brief, constructivist, curriculum-integrated EI elective led to significant improvements in EI knowledge, self-reported EI, and learner satisfaction among Egyptian medical students, supporting the feasibility and value of systematic EI instruction in undergraduate medical curricula. These findings provide quasi-experimental evidence from the Middle East that EI competencies can be developed through targeted educational interventions.

**Supplementary Information:**

The online version contains supplementary material available at 10.1186/s12909-026-09408-z.

## Introduction

Salovey and Mayer initially defined Emotional Intelligence (EI) as a set of skills for identifying, comprehending, and effectively managing one’s own emotions as well as those of others, using the knowledge gained to guide one’s behavior [1]. To improve effective interpersonal effectiveness, Goleman later broadened the construct into five main domains: self-awareness, self-regulation, motivation, empathy, and social skills [2]. These frameworks emphasize that emotional intelligence (EI) is a set of competencies that can be developed through education, rather than a fixed trait. Emotional intelligence has been linked to professional performance in studies demonstrating its role in promoting resilience, teamwork, effective communication [3, 4], and stress reduction, all of which contribute to improved patient outcomes and performance and reduced burnout [5, 6].

From a humanistic perspective, EI promotes empathy and compassion. Emotionally intelligent physicians are better equipped to manage emotionally charged scenarios such as breaking bad news, making complex clinical decisions, and addressing patients’ needs in culturally and emotionally sensitive ways [7, 8]. These skills are particularly important in the Egyptian healthcare system, where socioeconomic disparities, limited healthcare resources, high healthcare expenses, and inflation create a stressful environment for both patients and medical professionals. Additionally, Egypt’s cultural diversity requires physicians to adapt their communication to provide equitable, culturally sensitive care [9–11]. The integration of EI into curricula is supported by practical necessity and strong educational theory, particularly constructivism, which emphasizes that learners actively build meaning through interaction, reflection, and experimental activities [12]. This approach is further validated by the Accreditation Council for Graduate Medical Education (ACGME), which identifies EI skills as critical competencies for physician training [13].

Despite these benefits, EI has received little attention in medical education across Egypt and the broader Middle East [14], where training continues to prioritize technical competence over interpersonal development, with limited structured opportunities for students to develop and practice emotional intelligence skills systematically, risking the production of graduates less prepared to provide patients with adequate humanistic care [15, 16]. Egyptian medical students also train within a higher education system that is implementing large-scale enterprise resource planning (ERP) and other digital reforms, including AI-powered tools and generative AI platforms, while still facing structural and resource constraints and high patient loads, which further intensify the pressures on learners [17–19].In parallel, there is a well-documented decline in empathy among medical students, particularly after the third year when clinical duties increase and meaningful personal interactions with patients often decrease, so that, although students become more competent in diagnostic and procedural tasks, they may feel less able to engage with patients’ emotional needs. This divergence contributes to the perception that technical proficiency often surpasses emotional proficiency in real patient encounters, leaving graduates underprepared for the relational and emotional challenges of practice in the Egyptian context [20, 21]. By introducing a structured EI elective at the preclinical–clinical interface, our study seeks to address this decline in empathy directly, reinforcing self-awareness, perspective-taking, and emotion regulation at a time when students are particularly at risk of prioritizing efficiency over human connection.

### Literature review and research gaps

While a growing body of research in Egypt confirms that emotional intelligence (EI) is a significant predictor of success, linked to academic resilience in students [22] and to job satisfaction and performance among faculty [23], three critical gaps persist in the literature that this study explicitly addresses:

1. Methodological Gap: Most existing EI research in Middle Eastern medical education relies on correlational studies or cross-sectional designs that describe relationships at a single point in time, with limited quasi-experimental evidence demonstrating the causal impact of structured EI interventions on measurable learning outcomes in this cultural context [22, 24].
2. Educational Framework Gap: When EI has been addressed in education, both globally and nationally, interventions have often consisted of isolated workshops or short courses rather than integrated, longitudinal curriculum components [15, 16]. Although targeted interventions, such as Ramadan et al.'s workshop [25], demonstrate that EI is teachable, there is insufficient evidence of systematic, curriculum-integrated EI training evaluated using established educational assessment models (like Kirkpatrick's framework) in Egyptian medical schools.
3. Practical Implementation Gap: Although EI's importance is acknowledged, there is a lack of systematic integration into professional training, with limited evidence-based guidance on how to structure, deliver, and evaluate EI curricula within existing medical education programs in resource-limited settings [14, 16].

### Theoretical and evaluation framework

This quasi-experimental study bridges the gap between theoretical EI concepts and professional practice by providing the first systematic evaluation of a structured, curriculum-integrated EI elective for third-year students in an Egyptian medical school, using Kirkpatrick’s established framework, one of the most widely used methods for assessing educational interventions in health professions education [26, 27]. Levels 1 (Reaction) and 2 (Learning) are particularly relevant for early curriculum innovations and pilot courses, focusing on learner satisfaction and changes in knowledge, skills, and attitudes that can be feasibly measured in undergraduate settings using validated tools and pre–post evaluations [28, 29].In alignment with this framework, EI-related knowledge in this study is assessed through a blueprinted multiple-choice test mapped to the course learning outcomes, self-reported EI is measured using the Schutte Self-Report Emotional Intelligence Test (SSEIT) [30], and learner reactions are gathered via the Students’ Evaluation of Teaching (SETs) survey and qualitative feedback. These measures collectively enable the examination of how EI training translates into specific educational outcomes, knowledge gains, changes in SSEIT scores, and student satisfaction within an Egyptian undergraduate context.

### Aim of the study

This study aimed to: (1) Evaluate learners’ satisfaction with the course through the Students’ Evaluation of Teaching (SETs) survey; (Kirkpatrick Level 1); (2) Estimate the effect of the course on students’ EI-related knowledge and self-reported EI, as measured by a blueprinted knowledge test and the Schutte Self-Report Emotional Intelligence Test (SSEIT); (Kirkpatrick Level 2) and (3) Explore students’ qualitative feedback on the elective to inform the refinement of future EI curricula in undergraduate medical education.

### Research significance

This study provides important theoretical, educational, and contextual contributions to medical education in Egypt and the broader Middle East. It moves beyond correlation to causation by offering a concrete, low-cost model for integrating EI into an already crowded undergraduate curriculum, aligned with national academic reference standards and evaluated through a recognized framework (Kirkpatrick Levels 1 and 2). This delivers practical guidance for curriculum designers and faculty aiming to systematically develop humanistic and interpersonal skills.

The rest of this paper first describes the EI elective and study design, then presents the quantitative and qualitative results, and finally discusses their implications for EI curriculum development and the study’s limitations.

### Study design and setting

**Fig. 1:**
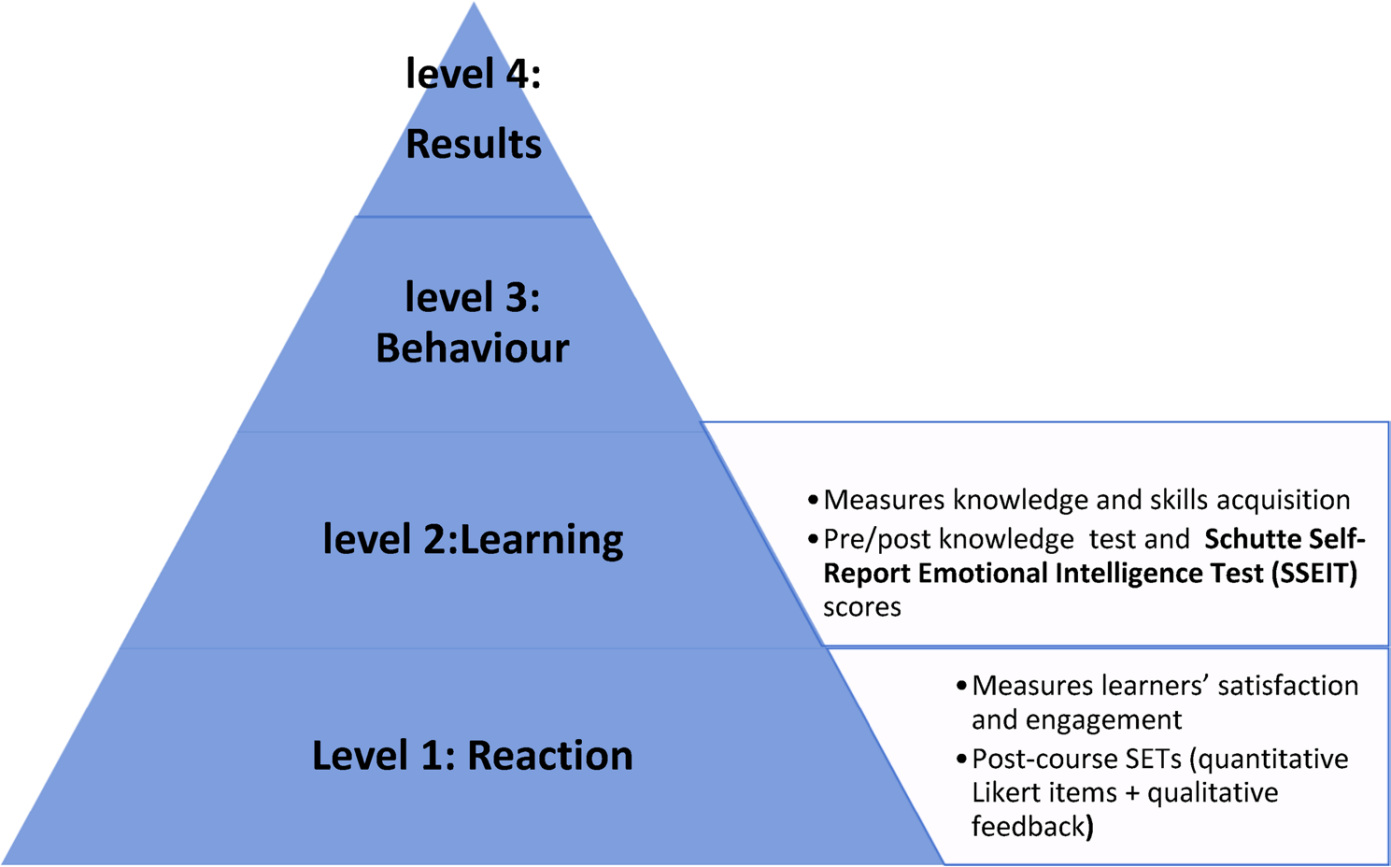
Application of Kirkpatrick model levels in the emotional intelligence elective course evaluation

This quasi-experimental study was conducted among third-year medical students enrolled in the Extended Modular Program (EMP) at the Faculty of Medicine, Ain Shams University, Cairo, Egypt, during the 2023–2024 academic year. The EMP delivers all core content of the general medical program through enhanced teaching methodologies, small-group instruction, integrated learning, and comprehensive assessment strategies. As a core curriculum component, students must complete 3 credit hours of elective courses during the pre-clerkship phase. The program offers a variety of courses each academic year to enrich students’ educational experience and develop their personal and professional skills. The evaluation framework for the EI course was based on the first two levels of Kirkpatrick’s model (Fig. 1).

### Sampling and recruitment

**Fig. 2:**
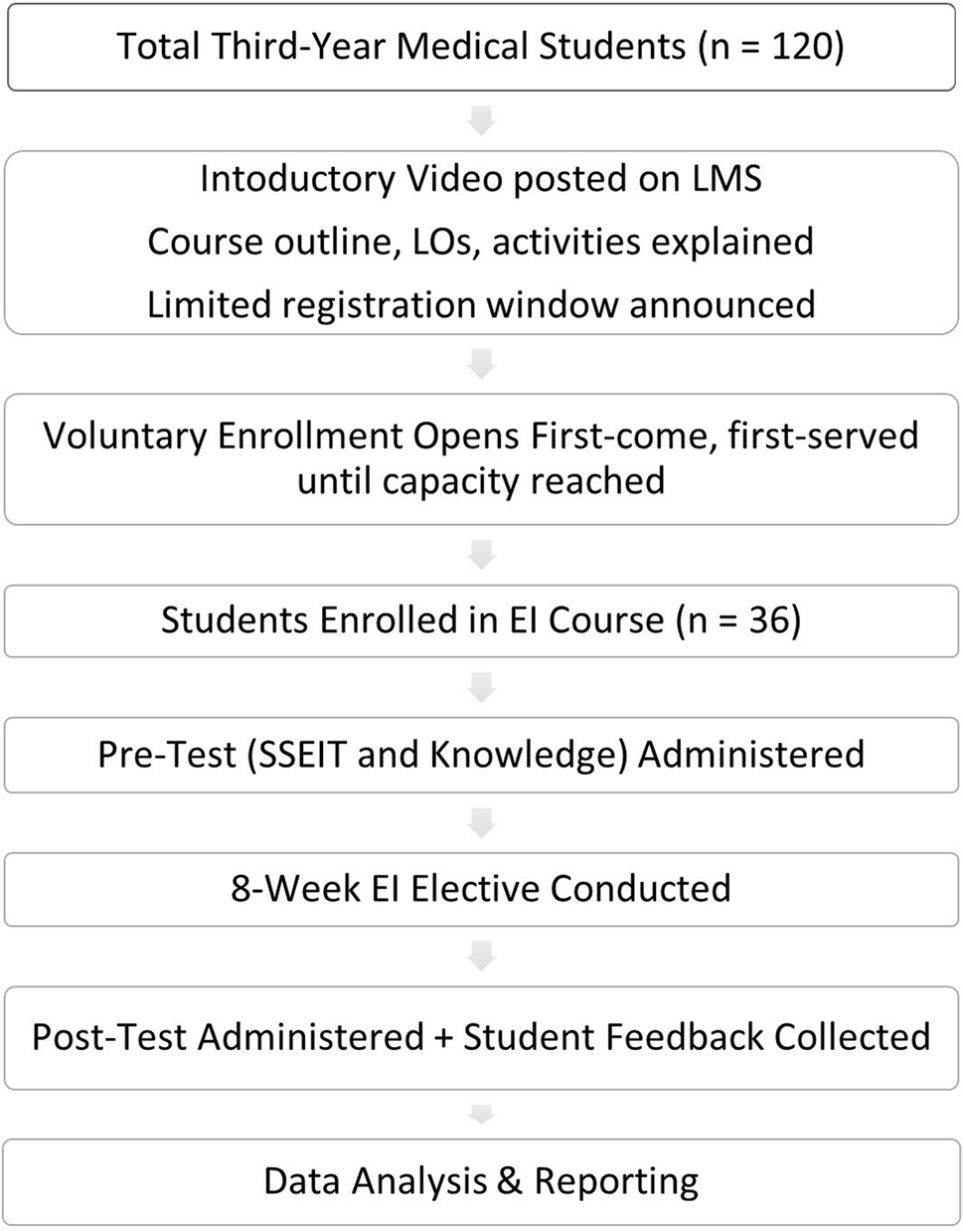
Process flowchart of EI elective enrollment and evaluation

The study cohort was drawn from the third-year medical class (EMP), which enrolls approximately 120 students annually. As part of the curriculum, students must select one elective from a pool of five options, with a maximum of 40 students per course to ensure small-group, interactive learning. The course instructor created an introductory video outlining the course, learning outcomes, and planned activities, which was uploaded to the university’s Learning Management System (LMS). The video was made available to all 120 third-year medical students, accompanied by a clear announcement about the opening of registration and its time-limited window. Students were invited to register voluntarily on a first-come, first-served basis until the maximum capacity was reached. Enrollment in the Emotional Intelligence elective was therefore capped at 40 students; ultimately, 36 students voluntarily registered, completed the course, and consented to participate in the study (Fig. 2). This “single-group pre-test/post-test” approach is a recognized and appropriate starting point for evaluating novel curriculum interventions in health professions education before moving to larger, multi-site randomized trials [28]. In practice, selecting a control group from students enrolled in different electives posed a significant risk to data integrity, as those students had no established educational relationship with the primary investigator. A low response rate and “survey fatigue” were anticipated, with no leverage on them, which would have compromised the statistical power of the comparison. By focusing on a single cohort, we leveraged the established teacher-student rapport to ensure a 100% completion rate and high-quality reflective data.

### Course intervention

The study author designed and delivered an elective course titled “Developing Your Emotional Intelligence in Life.” It was implemented over 10 weeks, comprising 14 contact hours. Aiming to be interactive and student-centered, the instructor implemented the course using a variety of instructional techniques, including small-group discussions, role-playing exercises, scenario-based activities, reflective writing, multimedia presentations, and hands-on projects. The curriculum covered the five domains of emotional intelligence (EI), and the learning outcomes were mapped to the National Academic Reference Standards (NARS) - Medicine 2017, issued by the National Authority for Quality Assurance and Accreditation of Education (NAQAAE) (Table 1).

**Table 1:** The outline of course sessions, key learning outcomes, and the corresponding class activities

Session	Topic	Key Learning Outcomes	Class Activities
1	Introduction to Emotional Intelligence	• Define Emotional Intelligence • Differentiate EI from IQ • Identify the EI life impact • Explore Goleman’s five elements • Assess EI and facial cues	• Knowledge pretest • Schutte Self-Report Emotional Intelligence Test (SSEIT) • Facial expressions quiz
2	Welcoming Empathy	• Differentiate empathy/sympathy/compassion • Recognize the importance and impact of daily practice • Assess individual empathy level	• Empathy questionnaire with immediate scoring
3	Empathy and social skills	• Identify how research described the unfairly excluded people from the empathetic community • Define the terms ageism and bullying • Reflect on the different types of bullying and related situations	• “Notes of Hope” reflection activity
4	The True, the Good, and the Beautiful	• Identify emotional dimensions of art and beauty • Reflect on emotional expression through art	• Origami art activity (“inflated heart”) and art appreciation exercises
5	Self-regulation	• Identify the six aspects of empathy • Discuss situations regarding emotional regulation • Reflect on the impact of social media on empathic relations	• Words quiz • Open discussion of *Whiplash* movie themes and empathy-related reflections
6	Self-Awareness	• Identify the four aspects of the Johari window for self-development and awareness. • Discuss how to apply these aspects in daily life • Identify motivational 30-day challenge ideas • Demonstrate awareness of culturally sensitive situations	• Johari’s traits activity • Discussion of a movie scene about vitiligo and stigma
7	Self-Expression and Integration	• Practice collaborative learning and creative self-expression	• Students’ presentations in diverse formats (video, poetry, art, etc.) based on the course’s inspiration

### Evaluation framework

Electronic questionnaires were distributed to participating students after they had provided their informed consent. The evaluation consisted of the following components:

#### Socio-demographic Profile

Students were asked to specify their age, gender, birth order, state of origin, and type of high school education. 

#### Course evaluation – Kirkpatrick model

The course was evaluated using the first two levels of Kirkpatrick’s model. Level 1 (Reaction) was assessed after the course using the Students’ Evaluation of Teaching (SETs) survey. This tool employs a five-point Likert scale covering course content, learning activities, instructor performance, and overall satisfaction, along with three open-ended questions for qualitative feedback. The instrument’s content validity was reviewed by three medical education experts, focusing on educational content, language, and cultural background. Items with an Item-Level Content Validity Index (I-CVI) ≥ 0.78 were retained; an overall Scale-Level CVI (S-CVI) ≥ 0.80 was considered strong; and items that did not meet these standards were revised or removed. For Level 2 (Learning), students’ learning was assessed using two measures: a 22-item knowledge test (multiple-choice and true/false items) and the Schutte Self-Report Emotional Intelligence Test (SSEIT), a 33-item validated instrument evaluating four EI domains (emotion perception, self-regulation, empathy/social skills, and emotion utilization).

#### Knowledge test

We prepared a 30-item knowledge test (20 MCQ and 10 True/False) to measure students’ cognitive understanding of emotional intelligence concepts before and after the elective course. A systematic blueprinting procedure was used to ensure the test’s content validity. We ensured the assessment encompassed a range of cognitive domains beyond recall by mapping each course outcome to Bloom’s taxonomy levels. The blueprint specified the proportional representation of the EI’s five domains across the test items. It included scenario-based questions that required the use of EI concepts to reduce random guessing. Three subject matter experts in medical education and psychiatry independently reviewed the blueprint and draft questions to ensure clarity, alignment, and cultural suitability. We removed or revised items with an item-level content validity index (I-CVI) below 0.78. The final stage involved testing the test with two senior medical students who were not part of the study group to assess clarity and duration [31]. Based on expert advice and student input throughout the trial, the test was shortened from 30 to 22 items (16 MCQ and 6 True/False). This reduction was intended to minimize student stress and reduce the likelihood of skipped questions due to fatigue or test length. The final version demonstrated strong content validity (S-CVI > 0.80) and covered all the course goals. It also struck a good balance between practicality and learner acceptance, which improved both reliability and validity.

#### Schutte Self-Report Emotional Intelligence Test (SSEIT)

The test was used to assess students’ emotional intelligence before and after the intervention (elective course). It is a reliable and valid tool selected for its strong psychometric properties and alignment with the Salovey and Mayer four-branch model [30–33]. We used the original English version without modification. It comprises 33 items that evaluate four key domains: emotion perception, managing self-emotions, managing others’ emotions, and emotion utilization. The test has demonstrated strong internal consistency across diverse populations, with Cronbach’s alpha values ranging from 0.85 to 0.90. Furthermore, it shows good test-retest reliability and acceptable construct validity over time, as evidenced by its positive correlation with related constructs, including empathy, social skills, and psychological well-being. It is a suitable tool for clinical research and educational settings due to its features and ease of use. Responses are recorded on a 5-point Likert scale, including three reverse-scored items (items 5, 28, and 33). Typical mean scores are around 124 (SD ≈ 13), with scores below 111 or above 137 considered unusually low or high [33, 34].

The internal consistency of the study instruments was examined in the current sample using Cronbach’s alpha coefficients.The knowledge test showed moderate reliability at baseline (Cronbach’s α = 0.63) and acceptable reliability after the intervention (Cronbach’s α = 0.74), and the Schutte Self-Report Emotional Intelligence Test demonstrated good reliability (Cronbach’s α = 0.77).

### Control measures for external confounding factors

To enhance study accuracy and minimize potential confounding from simultaneous exposure to other learning sources, several control measures were implemented: (1) Pre-test assessments (SSEIT and knowledge test) were administered immediately before the course began to establish an accurate baseline and minimize the influence of recent external EI-related exposure; (2) The knowledge test assessed course-specific concepts (Goleman’s five domains, Johari Window, empathy differentiation techniques) rather than general EI knowledge available through media or casual reading. The study’s focus on unique pedagogical approaches (origami exercises, course-specific movie analyses) created distinctive learning markers unlikely to be replicated through incidental exposure to television programs, specialist consultations, or other external sources; (3) Post-intervention assessments were conducted immediately upon course completion (within one week) to capture learning gains directly attributable to the intervention before external factors could significantly influence results; (4) All participants were third-year medical students from the same program (EMP) with similar academic backgrounds and concurrent coursework, which reduced variability in external learning exposures. (5) To maximize responses while maintaining professional and ethical research standards, micro-incentives were used to keep students engaged and create a low-stress learning environment. These incentives were offered as gestures of hospitality (edible reinforcers) rather than as coercive tools. Incorporating data collection into the curriculum (using the last 10 min of each session for surveys) and employing an anonymous “completion code” system helped ensure high participation rates. This approach allowed students to receive their Certificates of Completion for professional development without revealing their identities or the accuracy of the data.

### Data collection and analysis methods

Pre- and post-intervention data were collected anonymously using unique student codes and analyzed with SPSS version 27. Descriptive statistics summarized the characteristics of participants. The Shapiro-Wilk test confirmed normality; accordingly, parametric tests were used. Pre- and post-intervention scores on the knowledge test and SSEIT were compared using paired t-tests, and the size of the change was measured with Cohen’s d, interpreted as minimal (≤ 0.2), medium (0.2–0.5), or large (≥ 0.8). The Agreement Index (AI) was calculated from Likert-scale responses to measure student satisfaction, with 1.00–1.49 = Strongly Disagree and 4.50–5.00 = Strongly Agree. Changes in knowledge at the item level were assessed with McNemar’s test (*p* < 0.05 considered significant). Qualitative feedback from open-ended SET responses was thematically analyzed using inductive coding. The themes identified reflected students’ views on the learning experience, personal growth, and course design.

## Results

Using the Kirkpatrick model, the study evaluated the impact of an emotional intelligence (EI) elective course on third-year medical students, focusing on learner satisfaction (Level 1) and knowledge/skill development (Level 2).

### Participant demographics

The mean age of the cohort (*N* = 36) was 20.47 years. Twenty-four were female (66.6%), and twelve were male (33.3%). Twenty-four had IGCSE education (66.7%), while 5 had American Diplomas and 5 had Egyptian high school certificates. One had graduated from the International Baccalaureate, and the other had a Bac France. Half were firstborn. Most residents (66.7%) were based in Cairo before joining the college, while 25% resided outside Egypt.

### Kirkpatrick’s evaluation model, level 1 (Learners’ Satisfaction)

Ninety-eight percent of respondents to the Students’ Evaluation of Teaching (SETs) survey agreed that the instructor was well prepared, and 94% stated that the course material was current. The overall Agreement Index (AI) of 4.9/5 further demonstrated the high level of students' satisfaction with the course’s structure and delivery. (Table 2)

**Table 2:** Summary of students’ evaluation of the emotional intelligence elective course (SETs Survey, *N* = 36)

Domain	Survey Items	Average score (%)	Overall Agreement Index (AI)
Instructor	The instructor’s preparedness, interest in helping students, active, engaging teaching methods, using practical examples, and giving constructive feedback	96%	4.8
Course Content	The lectures, activities, and assignments complemented each other, increasing their knowledge, up-to-date, and well-organized	91%	4.5
Course Activities	The activities were clearly explained, useful, and had appropriate timing regarding the content	94%	4.7
Course Structure	The course duration, location, and environment were satisfying, gave confidence for more advanced work, and measured students’ abilities	90%	4.5
Overall Satisfaction	The course met students’ expectations, and would highly recommend it to other students	92%	4.6

Average score: of strongly agree and agree

*AI* Agreement Index based on a 5-point Likert scale (1 = Strongly Disagree, 5 = Strongly Agree)

### Qualitative feedback

The analysis of open-ended questions highlighted the positive impact of the interactive activities (such as the Johari Window, Hope notes, and origami exercises) and appreciation for the instructor’s teaching methods. It also included suggestions for future course improvement, such as incorporating clinical scenarios and placing greater emphasis on bullying scenarios (Table 3).

**Table 3:** Thematic analysis of students’ feedback on the emotional intelligence course

Main Theme	Sub-Theme	Description	Representative Quotes
1. Learning Experience	Interactive Engagement	Students most enjoyed hands-on activities that increased their emotional and cognitive engagement, such as “Hope Notes,” “Johari Window,” and “Origami Heart.”	*• The most interesting part was the activities; I wish to increase the time with more interactive ideas. “(S3*,* female)* *• “Analyzing the emotional themes in the movie makes the movie more vivid.” (S21*,* female)*
	Instructor’s Delivery	The instructor was often commended for her enthusiastic and imaginative approach, which incorporated storytelling, music, and art.	*Dr. Rania’s enthusiasm and personal stories had a significant impact on the delivery of these brainstorming topics. (S14*,* female)*
2. Personal Development	Self-Reflection	Activities like SSEIT test results and the Johari Window helped students understand their emotional patterns and blind spots.	*• The Johari window opened my eyes to the idea of the blind spot in our characters (S34*,* female)* *• “Now I understand the great effort needed for emotional regulation and its impact on my performance.”(S12*,* male)*
	Empathy and Perspective-Taking	Videos and media analysis helped increase their self-awareness, enabling them to distinguish between empathy, sympathy, and compassion.	*I discovered during the empathy session that I frequently mixed empathy with compassion. (p28*,* male)* *• “Discussing the final scene of the movie Whiplash made me think about my own goals in life.“(S6*,* male)*
3. Practical Implications	Clinical Relevance	Some students wanted the practical implications of emotional intelligence in medical practice, particularly in clinical communication.	*• “The medical experience stories I heard from my colleagues made me reconsider what kind of doctor I want to become.”* *(S10*,* male)*
4. Course Logistics	Duration and Structure	Some students thought that the sessions were too lengthy or not at the right time (such as late in the day); others recommended making some of the content more straightforward or reducing the length of the questionnaires.	*• “Some sessions felt overly long*,* especially after a full day of lectures. (S32*,* male)*
	Improvement Suggestions	The suggestions included enhancing the range of activities, incorporating more information on empathy in medicine, and introducing group therapy-style sessions.	*• “What about a monthly group discussion that could help us de-stress and feel more supported?”(S1*,* male)*
5. Learning Environment	Safe and Supportive Atmosphere	The welcoming atmosphere, freedom of speech, and chances for deep networking and friendship-building were valued by the students.	*• “The course helped me see new sides of my friends—and make new ones too.”(S10*,* female)*

*S* student

### Kirkpatrick evaluation model level 2 (The learning)

#### Knowledge test

Table 4 compares post-intervention scores with the pretest, showing a significant increase (*p* < 0.001), with mean scores rising from 11.81 (± 2.41) to 14.42 (± 2.41), and a large effect size (Cohen’s d = 0.94). It also highlights the items that showed significant change, using the McNemar test, indicating an improvement in understanding EI concepts in different situations, with no significant changes in topics related to bullying and emotional regulation.

**Table 4:** Pre-and post-analysis of knowledge acquisition: overall scores and item-level gains (Kirkpatrick Level 2 – Learning Evaluation)

Total score of tests	Pre Mean ± SD	Post Mean ± SD	t *p*-value
	11.81 ± 2.41	14.42 ± 2.41	5.63 <0.001
	**Estimate**	**95% CI**	**Significance**
Effect size Cohen’s *d*	0.94	1.33–0.54	S
Domain / Conceptual Area	Pre % Correct	Post % Correct	Significance (McNemar)
Definition & nature of EI	56–67%	67–78%	NS (all items)
Application of EI in a social context	66.7%	100%	S ***(p* = 0.001)**
Critical thinking in EI situations	72–89%	72–100%	NS (all items)
Interpretation of emotions	30.6–75%	61–86%	S (***p* = 0.012**, one item) NS (others)
Decision-making & conflict handling	25–36%	28–50%	NS (all items)
Bullying knowledge & attitudes	61–100%	58–100%	NS (most items)
Core EI components	19–61%	22–64%	NS (all items)
Predictors of performance	38.9%	77.8%	**S** (***p* = 0.002)**
Communication skills	50.0%	80.6%	**S** (***p* = 0.012)**
Johari Window	2.8%	44.4%	**S** (***p* < 0.001)**

*CI* confidence interval

*p* > 0.05: Nonsignificant (NS); Bold values indicate statistical significance (*p* < 0.05): Significant (S)

#### Schutte Self-Report Emotional Intelligence Test (SSEIT)

Post-test scores improved across all EI domains: perception of emotions (36.78 to 38.22), self-regulation (32.72 to 33.97), empathy/social skills (29.47 to 30.39), and utilization of emotions (23.58 to 24.22), with a total score increase from 122.36 to 126.69 (*p* < 0.001; Cohen’s d = 2.03). (Table 5).

**Table 5:** Participants’ scores in the schutte emotional intelligence test components, compared between pre- and post-tests, using a paired t-test

	Pre-test	Post-test	Paired t-test			Effect size Cohen’s d	
	Mean ± SD	Mean ± SD	T	*p* value	sig.	Estimate	95% CI
Perception of emotions	36.78 ± 5.19	38.22 ± 4.9	7.19	**< 0.001**	S	1.20	1.62–0.76
Managing one’s own emotions	32.72 ± 4.43	33.97 ± 4.42	6.35	**< 0.001**	S	1.06	1.46–0.64
Managing others’ emotions	29.47 ± 4.42	30.39 ± 4.47	5.87	**< 0.001**	S	0.98	1.37–0.57
Utilization of emotions	23.58 ± 3.08	24.22 ± 2.88	5.03	**< 0.001**	S	0.84	1.22–0.45
Total score of tests	122.36 ± 11.41	126.69 ± 10.7	12.16	**< 0.001**	S	2.03	2.6–1.45

*CI *confidence interval

*P* > 0.05: Nonsignificant (NS); Bold values indicate statistical significance (*p* < 0.05): Significant (S)

### Summary of students’ reflective writing topics

For the final assignments, “reflective tasks,” students choose from a variety of creative media, such as books, films, PowerPoint presentations, poetry, animations, narrated experiences, and videos. They represented different themes such as personal experiences with illness, trauma, or clinical encounters, and the psychological effects of social media and bullying. Several students used an artistic approach to exploring ideas about empathy, communication, and emotional intelligence by analyzing movie scenes. They also discussed issues in the healthcare system, including the psychological impacts of years of medical school, misdiagnosis, and professional hierarchy.

## Discussion

This quasi-experimental study evaluated a structured EI elective for third-year medical students in Egypt, using the Kirkpatrick evaluation model, a widely recommended framework for evaluating educational interventions in healthcare education [35]. The course was evaluated at the first two levels of the model: learners’ reactions (satisfaction) and learning outcomes (knowledge and skill development). A mixed-methods approach was employed, including a satisfaction survey, pre- and post-knowledge tests, and the Schutte Self-Report Emotional Intelligence Test (SSEIT). The results show that the course was highly satisfactory (Level 1) and caused notable improvements in both EI knowledge and self-reported EI skills (Level 2).

### Key findings vs. literature

The high level of students’ satisfaction (Agreement Index = 4.9/5) highlights the value of interactive, constructive, and learner-centered approaches in EI training. Students particularly valued activities such as the Johari Window for uncovering blind spots in self-awareness, along with reflective writing and creative exercises, including origami heart reflections and notes of hope, which offered joyful outlets for emotional expression. Role-playing and open discussions of everyday, often-overlooked situations allowed students to practice empathy and emotional regulation in a safe, structured environment. This preference for experiential learning and reflective teaching methods over passive lectures is well supported in the literature for fostering emotional growth [36, 37] and improving both EI and professional skills [38, 39]. The instructor’s energetic teaching style was appreciated, especially for the effective use of storytelling, multimedia integration, and real-life clinical scenarios, which heightened engagement and made abstract EI concepts more relatable. This aligns with studies that link facilitator clinical expertise and approachability to immediacy behaviors in practical soft skills training [40–42].

A notable finding from the qualitative feedback was students’ desire for more clinical application, including training on managing patient anger and delivering bad news. This indicates that the course provided a solid foundation, encouraging students to apply these skills in clinical settings. Therefore, our findings support an integrated approach for future EI training that combines active patient-centered activities [43], relatable pedagogical methods, and authentic emotional modeling to connect emotional intelligence theory with real-world clinical practice [44, 45].

The significant increase in knowledge test scores (*p* < 0.001) confirms that students successfully acquired core EI concepts and showed significant improvements across all four SSEIT domains: emotional perception, self-regulation, empathy, and emotion usage. Despite general gains in EI knowledge, the bullying-related items did not display a statistically significant change between pre- and post-test, a pattern consistent with prior research in Egyptian and international medical schools [46, 47]. Medical students often perceive verbal aggression, public criticism, and subtle exclusion as “discipline” rather than bullying [46–49]. Consequently, many participants may have started the course with relatively low baseline scores on bullying items, and a brief intervention was not sufficient to alter these deep-seated beliefs in a way detectable on a short knowledge test [46, 47]. Overall, these findings suggest that knowledge about bullying is more resistant to short-term change, and future curriculum improvements should include explicit, behaviorally anchored examples of bullying, ongoing reinforcement, and guided reflection on the hidden curriculum and power dynamics in medical training, to help reshape students’ implicit definitions of bullying [47–50].

The significant increase in total SSEIT scores following the intervention (*p* < 0.001) supports previous studies that show measurable benefits from targeted emotional intelligence interventions [51, 52]. The large effect size (Cohen’s d = 2.03) indicates an improvement in students’ self-perceived emotional intelligence, suggesting that even brief training can achieve meaningful results [53], especially for third-year medical students facing demanding workloads and complex patient interactions [54, 55]. However, this level of significance could partly be due to testing effects, such as becoming more comfortable with the questionnaire or social desirability bias after taking a highly rated course. Therefore, the results should be interpreted carefully, especially given the notable improvements in self-assessed emotional skills. Several studies on EI training have shown increased self-awareness, practical behavioral changes [56], better academic achievement, and reduced stress levels [57]. Moreover, EI-centered programs have successfully improved coping strategies, fostered supportive learning environments [58], and are vital for training physicians to interact effectively in interdisciplinary teams with empathetic communication skills [59].

The primarily female, self-selected composition of the cohort may have contributed to the increased involvement and positive outcomes, as prior research shows that female students generally display greater baseline empathy and emotional awareness compared to male students [60, 61]. They also outperform on tasks that require both cognitive and emotional skills [62, 63]. These gender-related differences likely stem from both biological and sociocultural factors. For example, women are typically educated to be more attuned to others’ feelings and needs [64]. While this does not alter the results, it highlights the importance of inclusive EI training that keeps all learners engaged. Future course iterations will focus on strategies to actively involve male students, such as emphasizing clinically relevant emotional intelligence scenarios (e.g., managing patient anger, handling critical feedback, leading ward rounds), problem-based tasks linking emotional intelligence to team performance and patient safety, and incorporating mixed-gender small groups and male role models who explicitly present emotional intelligence as a core professional skill for all physicians.

In conclusion, this study provides strong evidence that structured EI training should be integrated into medical education starting from pre-clinical years and reinforced during clinical rotations to address the well-documented decline in empathy after the third year [65, 66]. By bridging this gap through EI integration into medical practice, such as through longitudinal mentorship during clerkships [67] or simulated patient encounters [68], we can produce physicians who excel in both technical skills and emotional intelligence.

### Implications and curriculum integration

To translate the intervention’s benefits into practice, we propose a vertically integrated spiral curriculum to embed EI into medical education (Table 6). This paradigm introduces core EI skills early, reinforces them throughout training, and applies them in clinical settings, thereby preparing graduates for patient-centered care, effective interdisciplinary teamwork, developing self-awareness, and emotional regulation. Furthermore, it empowers educators to foster supportive learning environments that enhance student communication and empathy and improve medical students’ emotional awareness [69].

**Table 6:** Proposed framework for integrating emotional intelligence into medical education

Phase 1: Basic Emotional Intelligence Training (Years 1–2)		
**Content**	**Methods**	**Assessment**
The fundamental Emotional Intelligence (EI) domains of self-awareness, self-regulation, empathy, and motivation	• Art-based meetings per month (such as creating origami or drawing emotions) • Monthly sessions • Journaling feelings	• SSEIT and other self-reporting tools • Peer-reviewed reflections

Phase 2: Clinical application of emotional intelligence (Years 3–4)		
**Content**	**Methods**	**Assessment**
Emotional intelligence as a communication skill (e.g., active listening, delivering bad news)	Teaching techniques that use emotional intelligence include: • Video debriefs • Structured role-plays • Clinical storytelling • Simulated patient encounters	• Scenario-based multiple-choice questions • Communication rubrics • Faculty observation • Proactive listening and breaking bad news.

Phase 3: Emotional Leadership and Professionalism (Years 5)		
**Content**	**Methods**	**Assessment**
Interprofessional cooperation, conflict resolution, burnout management, and moral distress	• Longitudinal Mentorship • Reflective rounds	• 360-degree assessments • Mentoring OSCEs • Patients’ feedback
Cross-Cutting Elements		
• EI themes to be incorporated into modules on professionalism, ethics, and communication skills. • Including EI in student wellness initiatives to promote resilience. • Developing faculty to ensure that emotional intelligence behaviors are consistently modeled and reinforced		

In response to students’ requests for more clinical applications, especially in situations involving delivering bad news, managing angry patients, and handling emotionally charged scenarios, we planned to include these topics in Phases 2 and 3 of the proposed spiral curricula (clinical years). The EI concepts taught in the pre-clinical elective will be reviewed and practiced through structured role-plays, simulated patient encounters, and video debriefings that focus on the requested situations, including addressing complaints and resolving conflicts within the healthcare team. These activities will be integrated into the existing communication and clinical skills modules. Students will use standardized patients, checklists, and focused feedback to help connect their EI skills to specific clinical behaviors. Additionally, we proposed small-group reflection sessions after challenging clinical experiences to enhance learning by allowing students to examine real instances where emotional intelligence skills are tested and to develop alternative strategies for future situations.

The implications of this spiral EI curriculum also go beyond course design to influence institutional policy. The positive results of this pilot support the inclusion of EI-related competencies in institutional graduate attributes and program learning outcomes, providing a strong rationale for shifting from small, optional electives to formally integrating EI components into the core undergraduate curriculum. The curriculum map and assessment framework should align with national accreditation and quality assurance standards (NARS Medicine, 2017), particularly in professionalism, communication, and student well-being, thereby making EI training a key criterion in program evaluation. Additionally, the model can guide faculty development policies through workshops that enable clinical teachers to demonstrate emotionally intelligent behaviors in wards and clinics, as well as student support initiatives like incorporating EI concepts into wellness and burnout prevention programs, ensuring a supportive institutional environment that reinforces the spiral curriculum.

### Limitations and future directions

Using a single-group pre–post design without a comparison group limits the ability to establish causality, as factors like maturation and concurrent learning could have affected the results. This design was a practical choice for an initial pilot within a single elective cohort, emphasizing the need for future studies involving multiple cohorts or institutions with comparison groups to strengthen causal claims. The generalizability of these findings is limited by the relatively small sample size (*n* = 36), recruitment from a single medical school, and the voluntary nature of participation in an elective course, which may have introduced self-selection bias and reduced the representativeness of the wider medical student population. Using self-reported measures of EI (e.g., SSEIT) can introduce social desirability bias and may not fully capture behavioral changes. Although the SSEIT demonstrated good internal consistency in the current sample, the knowledge test showed only moderate baseline reliability, suggesting that some items may require further refinement to improve the instrument’s stability in future implementations. Additionally, self-assessment tests may be influenced by participant fatigue, incomplete responses, or random guessing, and online response rates are likely to decrease. Baseline SSEIT scores were within typical ranges for university and medical students, and pre-test knowledge scores indicated room for improvement, suggesting that our findings warrant confirmation in larger, more diverse groups. Although control measures, such as reminders and incentives, were used to reduce external confounding factors, we recognize that complete isolation from all EI-related stimuli was not possible. However, the immediate pre-post design, content-specific assessments, and the focused intervention period lend reasonable confidence that observed improvements mainly reflect the structured course intervention rather than incidental learning.

Future research could enhance external validity by using larger, more diverse randomized samples across multiple institutions and cultures, including control or comparison groups where feasible. Additionally, while the short follow-up captures immediate learning gains, it does not assess the durability of EI improvements or their impact on clinical behavior and patient outcomes. Because post-intervention outcomes were measured immediately after course completion, the durability of these improvements remains uncertain. Future studies should incorporate delayed follow-up assessments several months after the intervention to determine whether gains in emotional intelligence knowledge and self-reported competencies are sustained over time.

For long-term follow-up through school and residency, future studies could adopt mixed methods designs or extend to the next two Kirkpatrick levels: behavioral application in clinical settings (level 3) and patient impact (level 4). This approach would provide a more comprehensive understanding of how EI training influences real-world professional performance and patient care in Egypt and the broader MENA region.

## Conclusion and recommendations

This quasi-experimental study showed that a structured elective on EI significantly enhanced third-year medical students’ knowledge of emotional intelligence and self-reported competencies, with extremely high learner satisfaction. These results provide strong evidence that EI is a teachable skill, and even a brief, targeted intervention can offer substantial educational advantages. The course promoted empathy, self-awareness, and emotion regulation skills essential for safe, compassionate, and effective medical practice through interactive, constructivist methods. The successful implementation and positive outcomes of this elective strongly support the systematic integration of EI training into undergraduate medical curricula. Future multicenter studies with longitudinal follow-up are recommended to evaluate the long-term retention of these skills and their influence on clinical behavior and patient care outcomes.

## Supplementary Information

Supplementary Material 1.

## Data Availability

The datasets used and/or analyzed during the current study are available from the corresponding author on reasonable request.
