# Supplementary material for: Enhancing emotional intelligence in Egyptian medical students: a quasi-experimental study using Kirkpatrick’s model
Source: BMC Med Educ. 2026 May 19;26:780. doi: 10.1186/s12909-026-09408-z (PMC13188492; doi:10.1186/s12909-026-09408-z)
Supplement: Supplementary file 1 — Supplementary Material 1. [file 12909_2026_9408_MOESM1_ESM.pdf]

Appendix (1)

Students' Evaluation of Teaching SETs)

	Strongly disagree	Disagree	Neither agree nor disagree	Agree	Strongly agree
**Instructor Assessment**	**1**	**2**	**3**	**4**	**5**
The instructor was well-prepared for the course					
The instructor showed an interest in helping students learn					
The instructor used active teaching methods and engaged the learners					
The instructor could respond to the ambiguities and questions of the learners					
The instructor used practical examples during teaching					
I received useful feedback on my performance during the course					
**Course content assessment**					
The lectures, activities, and assignments complemented each other					
The instructional materials increased my knowledge and skills in the subject matter					
The course content was up to date					
The course was organized in a manner that helped me understand the underlying concepts					
**Activities assessment**					
The activities were clearly explained					
The activities were useful					
The timing of activities was appropriate for the content					
**Course structure assessment**					
The course duration was satisfying to me					
The educational location and environment were satisfying					
The course gave me the confidence to do more advanced work in the subject					
The class activities and assignments measured my knowledge of the course material					

Overall satisfaction					
I would highly recommend this course to other students					
Overall, this course met my expectations					
Which activities did you find most useful? Why?					
What did you like most / least in the course?					
If you have any recommendations in addition to the above cases to increase the quality of the courses, please write them here					

Appendix (2)

MCQ Pre and post test

Item no.	Questions	Learning outcomes
1	**Which of the following is a primary characteristic of Emotional Intelligence (EI)?**<br>A. Advanced technical problem-solving proficiency<br>B. Enhanced quality of interpersonal relationships*<br>C. High aptitude for abstract cognitive reasoning<br>D. The ability to suppress anger<br>E. Unsure	Identify the 5 elements needed to enhance emotional intelligence
2	**True or False: A high Emotional Quotient (EQ) is a significant predictor of success in social dynamics and effective decision-making.**<br>A. True*<br>B. False<br>C. Unsure	Identify the impact of EI on life
3	**Which term describes the ability to anticipate and understand another person's emotions, even when they are not explicitly communicated?**<br>A. Empathy*<br>B. Sympathy<br>C. Compassion<br>D. Pity<br>E. Unsure	Describe the difference between empathy, sympathy, and compassion
4	**Based on the provided image, which emotion does this facial expression represent?**<br>a) Sadness<br>b) Shame<br>c) Disgust<br>d) Contempt*<br>e) Don't know<br>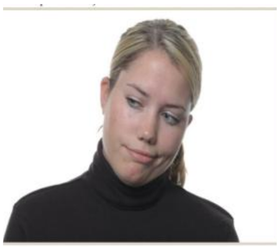	Explore his/her ability to detect facial expressions

5	**During a meeting, a colleague claims credit for your work. What is the most emotionally intelligent response?**<br>A. Publicly confront the colleague regarding the oversight.<br>B. Address the issue in a private, professional discussion post-meeting.<br>C. Remain silent to maintain group harmony.<br>D. Publicly acknowledge the colleague's points while professionally integrating specific details of your own contributions. *<br>E. Unsure	Develop a sense of understanding and expressing emotions in difficult situations in life.
6	**If a student receives a disappointing grade on a critical midterm, which response demonstrates high EI?**<br>A. Formulating and committing to a strategic academic improvement plan. *<br>B. Concluding that he/she lacks the aptitude for the chosen field.<br>C. Dismissing the course as inconsequential to their future.<br>D. Attempting to negotiate a grade change without merit.<br>E. Unsure	Develop a sense of understanding and expressing emotions in difficult situations in life.
7	**How should challenging or disagreeing with others' opinions be viewed within a collaborative environment?**<br>A. As a necessary component of robust problem-solving*<br>B. As inherently unprofessional or disrespectful<br>C. As acceptable only when one is objectively certain he/she is correct<br>D. As a detrimental action to be avoided<br>E. Unsure	Explore the problems that can arise when we don't know how to work with our empathic abilities.
8	**As a member of a diversity-focused organization, you overhear a peer tell a biased joke. What is the most effective intervention?**<br>A. Act neutrally and ignore the comment.<br>B. Discuss the behavior with the individual in a private setting later.<br>C. Address the comment immediately.*<br>D. Formally recommend that the individual undergo mandatory sensitivity training.<br>E. Unsure	Explore the problems that can arise when we don't know how to work with our empathic abilities.

**9**	**Based on the provided image, which emotion does this facial expression represent?**<br><br>a) Sadness<br>b) shame *<br>c) Embarrassment<br>d) pride<br>e) Unsure<br>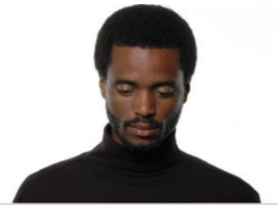	Explore his/her ability to detect facial expressions
**10**	**True or False: Bullying incidents typically occur in isolation, without the presence of peers.**<br>A. True<br>B. False*<br>C. Unsure	Identify the definition of bullying<br><br>Differentiate between the different types of bullying<br><br>Describe how to deal with different situations of bullying
**11**	**True or False: Engagement in bullying behaviors is statistically limited to males.**<br>A. True<br>B. False*<br>C. Unsure	
**12**	**True or False: The dissemination of rumors and social exclusion are classified as forms of bullying.**<br>A. True*<br>B. False<br>C. Unsure	
**13**	**True or False: Research indicates that most individuals who bully possess chronically low self-esteem.**<br>A. True<br>B. False*<br>C. Unsure	
**14**	**True or False: Victims of bullying should be encouraged to respond with physical retaliation to stop the behavior.**<br>A. True<br>B. False*<br>C. Unsure	
**15**	**True or False: Reporting a bullying incident to an authority generally exacerbates the situation.**<br>A. True<br>B. False*<br>C. Unsure	

16	**What is a primary distinction between Emotional Intelligence and traditional Cognitive Intelligence?**<br>A. EI is a static set of innate skills.<br>B. EI is more easily quantified via standardized testing.<br>C. EI emphasizes the integration of emotional reasoning into logic.*<br>D. EI is a subset of general IQ.<br>E. Unsure	Identify the impact of EI on life
17	**Which factor is the best predictor of academic performance?**<br>A. Trait Emotional Intelligence<br>B. Intelligence quotient (IQ)*<br>C. Specific Personality traits<br>D. Socioeconomic status<br>E. Unsure	Identify the difference between EI and IQ
18	**In the context of EI, what does "Self-Motivation" primarily entail?**<br>A. The internal drive to direct one's actions toward a goal.*<br>B. The capacity for physical perseverance.<br>C. The ability to mirror the emotions of others.<br>D. Maintaining a positive outlook regardless of reality.<br>E. Unsure	Identify the 5 elements needed to enhance emotional intelligence
19	**Which of the following best defines "Emotional Intelligence"?**<br>A. The reflexive feeling of others' emotions.<br>B. Being inherently "smart" in social settings.<br>C. The total control over one's anger.<br>D. The synthesis of emotional comprehension and social proficiency.*<br>E. Unsure	Define the term emotional intelligence

**20**	**What is the most accurate definition of "Interpersonal Skills"?**<br>A. The ability to suppress negative reactions during conflict.<br>B. The integration of personal awareness and social competence. *<br>C. The deep understanding of one's own internal feelings.<br>D. The singular ability to provide empathy to others.<br>E. Unsure	Identify the components of communication skills that impact social relationships
**21**	**In the context of interpersonal communication, which factor typically carries the most weight in message interpretation?**<br>A. Body language and non-verbal cues*<br>B. Vocal tone and inflection<br>C. Specific word choice<br>D. The intensity of the speaker's emotions<br>E. Unsure	Identify the components of communication skills that impact social relationships
**22**	**What is the primary objective of utilizing the Johari Window model?**<br>A. To improve self-regulation and emotional control.<br>B. To establish mutual agreements in professional negotiations.<br>C. To categorize different personality types.<br>D. To facilitate greater self-awareness and mutual understanding within groups. *<br>E. Unsure	Explore the problems that can arise when we don’t know how to work with our empathic abilities.

Appendix (3)

The Schutte Self Report Emotional Intelligence Test (SSEIT)

Instructions: Indicate the extent to which each item applies to you using the scale in the five columns to the right of each item.

Test Items	Strongly disagree	Disagree	Neither disagree	Agree	Strongly agree
1. I know when to speak about my personal problems to others	1	2	3	4	5
2. When I am faced with obstacles, I remember times I faced similar obstacles and overcame them	1	2	3	4	5
3. I expect that I will do well in most things I try	1	2	3	4	5
4. Other people find it easy to confide in me	1	2	3	4	5
5. I find it hard to understand the non-verbal messages of other people*	5	4	3	2	1
6. Some of the major events of my life have led me to re-evaluate what is important and not important	1	2	3	4	5
7. When my mood changes, I see new possibilities	1	2	3	4	5
8. Emotions are one of the things that make my life worth living	1	2	3	4	5
9. I am aware of my emotions as I experience them	1	2	3	4	5
10. I expect good things to happen	1	2	3	4	5
11. I like to share my emotions with others	1	2	3	4	5
12. When I experience a positive emotion, I know how to make it last	1	2	3	4	5
13. I arrange events others enjoy	1	2	3	4	5
14. I seek out activities that make me happy	1	2	3	4	5
15. I am aware of the non-verbal messages I send to others	1	2	3	4	5
16. I present myself in a way that makes a good impression on others	1	2	3	4	5
17. When I am in a positive mood, solving problems is easy for me	1	2	3	4	5

18. By looking at their facial expressions, I recognize the emotions people are experiencing	**1**	**2**	**3**	**4**	**5**
19. I know why my emotions change	**1**	**2**	**3**	**4**	**5**
20. When I am in a positive mood, I am able to come up with new ideas	**1**	**2**	**3**	**4**	**5**
21. I have control over my emotions	**1**	**2**	**3**	**4**	**5**
22. I easily recognize my emotions as I experience them	**1**	**2**	**3**	**4**	**5**
23. I motivate myself by imagining a good outcome to tasks I take on	**1**	**2**	**3**	**4**	**5**
24. I compliment others when they have done something well	**1**	**2**	**3**	**4**	**5**
25. I am aware of the non-verbal messages other people send	**1**	**2**	**3**	**4**	**5**
26. When another person tells me about an important event in his or her life, I almost feel as though I have experienced this event myself	**1**	**2**	**3**	**4**	**5**
27. When I feel a change in emotions, I tend to come up with new ideas	**1**	**2**	**3**	**4**	**5**
28. When I am faced with a challenge, I give up because I believe I will fail*	**5**	**4**	**3**	**2**	**1**
29. I know what other people are feeling just by looking at them	**1**	**2**	**3**	**4**	**5**
30. I help other people feel better when they are down	**1**	**2**	**3**	**4**	**5**
31. I use good moods to help myself keep trying in the face of obstacles	**1**	**2**	**3**	**4**	**5**
32. I can tell how people are feeling by listening to the tone of their voice	**1**	**2**	**3**	**4**	**5**
33. It is difficult for me to understand why people feel the way they do*	**5**	**4**	**3**	**2**	**1**
**Total score**					

Please note that items 5, 28, and 33 feature a reverse scale it's not a mistake!), where "Strongly disagree"= 5 and "Strongly agree" = 1.

Appendix (4)

Informed Consent in English and Arabic

Research Informed Consent

Title of the study:

Evaluating the Impact of an Emotional Intelligence Elective Course: A Quasi-experimental Study in an Egyptian Medical School

Principal Investigator:

Full Name: **Rania Moustafa Kamel Abdelbaset Moustafa Hadhoud**

Occupation / Specialty: Ass. Prof. of Forensic Medicine and Toxicology-ASU

Coordinator of students' development unit-MEDU-AFCM

Affiliation: AFCM -Medical Education Development Unit

Full mailing address: hadhoud.mee801batch2@gmail.com

Phone number:01223747656

Purpose of the study:

This study is designed to evaluate the effectiveness of the emotional intelligence elective course for third-year medical students at Ain Shams University, through evaluation of the students' satisfaction at the first level of the Kirkpatrick evaluation model (Reaction) and the related knowledge and skills after completing the course at the second level of the Kirkpatrick evaluation model (Learning).

Procedures:

A quasi-experimental study will involve a pretest and a posttest [O1 - X - O2] after the educational intervention. The study will be conducted in the Extended Modular Program (EMP) at the Faculty of Medicine, Ain Shams University, with 3rd-year medical students enrolled in the EI elective course. It will use purposive (selective) sampling of the total number of students attending the course.

A pre- and post-test will be done of 22 questions, including representative samples of the course topics and learning outcomes, to measure the level of knowledge, skills, and values acquired by the participants from the program, including scenario-based situations that require the application of knowledge to measure EI skills.

Students' Evaluation of Teaching SETs) The questionnaire will be distributed at the end of the course to determine the level of satisfaction of the participants or how they feel about the elective course, scored based on a 5-point Likert scale from strongly disagree to strongly agree.

The Schutte Self-Report Emotional Intelligence Test SSEIT): related to the three aspects of EI: Appraisal and expression of emotion, Regulation of emotion, and Utilization of emotion

It includes a 33-item using a 1 strongly agree) to 5 strongly disagree) scale for responses

Disadvantages for you:

No harm at all.

Benefits for you:

Self-evidence and satisfaction, as you will be sharing in a survey that would potentially affect the medical education and practice in Egypt.

Confidentiality:

Please do not write any identifying information.

Every effort will be made by the researcher to preserve your confidentiality, including the following:

•Assigning code names/numbers for participants that will be used on all research notes and documents.

•Keeping notes, interview transcriptions, and any other identifying participant information in a locked file cabinet in the personal possession of the researcher.

Voluntary participation:

Your participation in this study is voluntary. It is up to you to decide whether to take part. If you decide to take part, you will be asked to sign a consent form. After you sign the consent form, you are still free to withdraw at any time and without giving a reason. Withdrawing from this study will not affect the relationship you have, if any, with the researcher. If you withdraw from the study before data collection is completed, your data will be returned to you or destroyed.

Consent:

I have read and understand the information provided and have had the opportunity to ask questions. I understand that my participation is voluntary and that I am free to withdraw at any time, without giving a reason and at no cost. I understand that I will receive a copy of this consent form. I voluntarily agree to take part in this study.

Participant's Name ___________________________

Participant's Signature ______________________

Date _________

Appendix (5)

The informed consent Arabic Format

نموذج الموافقة على بحوث التعليم الطبي

عنوان البحث: تقييم أثر مقرر اختياري للذكاء العاطفي: دراسة شبه تجريبية في كلية طب مصرية.

الباحث الرئيسي: الاسم: ا.م.د/رانية مصطفى كامل مصطفى هدهود

التخصص: أستاذ مساعد الطب الشرعي والسموم – كلية الطب – جامعة عين شمس

منسق وحدة تطوير مهارات الطلاب – تطوير التعليم - كلية الطب بالقوات المسلحة

رقم الهاتف المحمول: 01223747656

المقدمة والغرض من الدراسة:

أصبح تعليم طلاب الطب ليصبحوا أطباء متعاطفين هدفًا تعليميًا واضحًا في التعليم الطبي وقد أظهرت الدراسات التي أجريت على طلاب الطب تراجع مستوى الذكاء العاطفي والتعاطف مع تقدم السنوات، خاصة بعد السنة الثالثة من كلية الطب. ويمكن تطبيق برامج تدريسية لذلك من خلال مسارات مناهج بديلة "مناهج اختيارية". وينظر الطلاب إلى المواد الاختيارية على أنها تجربة قيمة تحظى بتقدير كبير، ولها فوائد في توفير التعليم الأفضل والنجاح الأكاديمي. كمرحلة أساسية لتطوير المناهج والبرامج، هناك حاجة متزايدة لتقييم المناهج والبرامج التعليمية لأغراض مختلفة، ولكن عادة للنظر في تحقيق الأهداف. وفي التعليم العالي، أحد هذه النماذج التي يتم نقلها إلى تقييم البرنامج هو النموذج الذي اقترحه دونالد كيرك باتريك. وهو أحد النماذج الأكثر شهرة والأكثر استخدامًا لتقييم برامج التدريب والتطوير.

تهدف هذه الدراسة إلى تقييم مدى فاعلية المقرر الاختياري الذكاء العاطفي) لطلاب الطب بالسنة الثالثة بجامعة عين شمس، من خلال تقييم رضا الطلاب في المستوى الأول لنموذج تقييم كيرك باتريك (رد الفعل) وما يرتبط به من معارف ومهارات بعد الانتهاء من دورة المستوى الثاني من نموذج تقييم كيرك باتريك (التعلم).

مشاركتك طوعية:

مشاركتك طوعية تماما، لذا فإن الأمر متروك لك لتقرر ما إذا كنت ستشارك في هذه الدراسة أم لا. إذا كنت ترغب في المشاركة، سيطلب منك التوقيع على هذا النموذج. إذا قررت المشاركة في هذه الدراسة، فلا يزال بإمكانك الانسحاب في أي وقت ودون إبداء أية أسباب لقرارك.

إذا كنت لا ترغب في المشاركة، فلا يتعين عليك تقديم أية أسباب لقرارك بعدم المشاركة ولن يتأثر عملك والمزايا الأخرى التي يحق لك الحصول عليها بقرارك بالمشاركة أم لا.

ماذا تتضمن الدراسة؟

تصميم الدراسة

هذه دراسة شبه تجريبية تتضمن اختبار قبل المقرر ثم تنفيذ المقرر واختبار بعده.

إعداد الدراسة / مصادر البيانات:

سيكون مصدر البيانات عبارة عن:

- إجراء اختبار قبلي وبعدي مكون من 22 سؤال تتضمن عينات تمثيلية لموضوعات المقرر متماشيا مع مخرجات التعلم لقياس مستوى المعرفة والمهارات والقيم التي اكتسبها المشاركون من البرنامج، بما في ذلك المواقف المبنية على السيناريو والتي تتطلب التطبيق المعرفة لقياس مهارات الذكاء العاطفي.
- استبيان تقييم الطلاب للتدريس في نهاية المقرر لتحديد مستوى رضا المشاركين أو مدى شعورهم تجاه المقرر الاختياري الذي تم تسجيله بناءً على مقياس ليكرت المكون من 5 نقاط من لا أوافق بشدة إلى أوافق بشدة.
- اختبار شوت للتقرير الذاتي للذكاء العاطفي (SSEIT): يتعلق بالجوانب الثلاثة للذكاء العاطفي تقييم العواطف والتعبير عنها وتنظيم العواطف والاستفادة منها. يتضمن 33 عنصرًا باستخدام مقياس من 1 أوافق بشدة) إلى 5 لا أوافق بشدة) للإجابات

مجتمع الدراسة / المشاركون:

- مجتمع الدراسة: سيتم إجراء الدراسة في البرنامج الطبي الموسع EMP) - كلية الطب - جامعة عين شمس على طلاب الطب في السنة الثالثة الذين يتلقون المقرر الاختياري الذكاء العاطفي). وسيتم أخذ عينة انتقائية) على إجمالي عدد الطلاب الذين يحضرون المقرر

معايير الاشتمال:

- طلاب الطب في السنة الثالثة من المرحلة الجامعية الذين التحقوا طوعًا بالمقرر الاختياري وأتموه؛ والذين يوافقون على المشاركة في الدراسة

- معايير الاستبعاد:

طلاب الطب في السنة الثالثة من المرحلة الجامعية الذين يحضرون مقررات اختيارية غير مقرر الذكاء العاطفي أو الذين لا يوافقون على المشاركة في الدراسة.

ما هي المخاطر والمضايقات المحتملة للمشاركة؟

لا توجد أية مخاطر أو مضايقات محتملة للمشاركة في هذه الدراسة. لن يتم طرح أية أسئلة ذات طبيعة حساسة

ما فوائد المشاركة في هذه الدراسة؟

لا توجد فوائد متوقعة لك من المشاركة في هذه الدراسة. نأمل أن يتم استخدام المعلومات المستفادة من هذه الدراسة في المستقبل لإفادة تطوير التعليم الطبي.

ماذا يحدث إذا قررت سحب موافقتي على المشاركة؟

مشاركتك في هذا البحث طوعية تماما. يمكنك الانسحاب من هذه الدراسة في أي وقت. إذا قررت دخول الدراسة والانسحاب في أي وقت في المستقبل، فلن تكون هناك عقوبة أو خسارة في المزايا التي يحق لك الحصول عليها.

إذا اخترت الدخول في هذه الدراسة ثم قررت الانسحاب في وقت لاحق، سيتم إتلاف جميع البيانات التي تم جمعها عنك أثناء تسجيلك في الدراسة.

هل ستبقى مشاركتي في هذه الدراسة سرية؟

سيتم احترام سريتك. ومع ذلك، قد يتم فحص سجلات البحث التي تحدد هويتك بحضور الباحث الرئيسي لغرض جودة ودقة البحث. لن يتم نشر أي معلومات أو سجلات تكشف عن هويتك دون موافقتك، ولن تتم إزالة أو نشر أية معلومات أو سجلات تكشف عن هويتك دون موافقتك ما لم يكن ذلك مطلوبا بموجب القانون.

لن يتم نشر أية معلومات متعلقة بالبحث تخص هويتك بأي شكل من الأشكال لأنه تمت إزالة جميع معلومات التعريف بحيث تصبح المعلومات مجهولة المصدر ولا توجد إمكانية لربط هويتك بمعلوماتك.

بينما نشجع المشاركين على عدم مناقشة محتوى الاستبيان، لا يمكننا التحكم في ما يفعله المشاركون بالمعلومات التي تمت مناقشتها، ولا يمكن ضمان السرية

الإتـــاحة:

سيتم نشر البحث في أحد الدوريات المتاحة عبر الإنترنت على هيئة معلومات مجمعة ليستفيد منها الباحثون الآخرون، وبمجرد إتاحة البيانات عبر الإنترنت، لن يتمكن المشارك من سحب بياناته من الدراسة.

بمن أتصل إذا كانت لدي أسئلة حول الدراسة أثناء مشاركتي؟

إذا كان لديك أية أسئلة أو ترغب في مزيد من المعلومات حول هذا الجزء من الدراسة قبل أو أثناء المشاركة، يمكنك الاتصال بالدكتورة/ رانية مصطفى هدهود باحث رئيسي) على رقم الهاتف المحمول: **01223747656**

هذه الوثيقة هي نموذج الموافقة المستنيرة وليست عقدا، وبالتالي أنت لا تتخلى عن أية حقوق قانونية بالتوقيع عليها.

أنا أقوم بالتوقيع على هذا النموذج للإشارة إلى أنني قد قرأت وفهمت وأقدر المعلومات المتعلقة بالدراسة.

تحقق من موافقتك على القائمة الآتية:

- لقد أتيحت لي الفرصة لطرح أسئلة حول المعلومات المقدمة في نموذج الموافقة هذا وحصلت على ردود مرضية على أسئلتي.
- أفهم أن مشاركتي في هذه الدراسة طوعية وأنني حر تماما في رفض المشاركة أو الانسحاب من هذه الدراسة في أي وقت.
- أفهم أنني لا أتنازل عن أي من حقوقي القانونية نتيجة للتوقيع على نموذج الموافقة.
- لقد قرأت هذا النموذج وأوافق بحرية على المشاركة في هذه الدراسة.
- لقد قيل لي أنني سأستلم نسخة موقعة من هذا النموذج.

التوقيـــع:

توقيع المشارك	اسم المشارك	التاريخ
توقيع الباحث	اسم الباحث د/رانية مصطفى هدهود	التاريخ

Appendix (6)

The Sessions Photo Gallery

The Johary window activity	
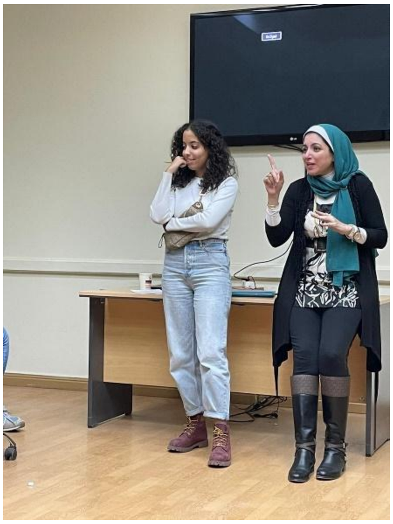	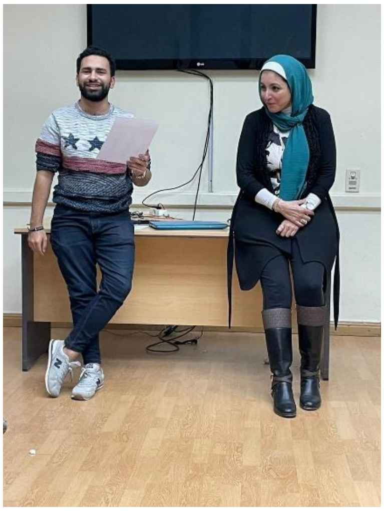
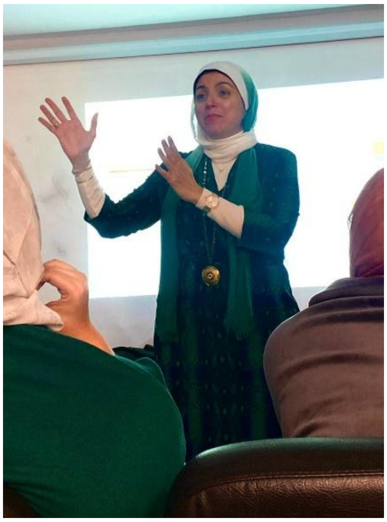	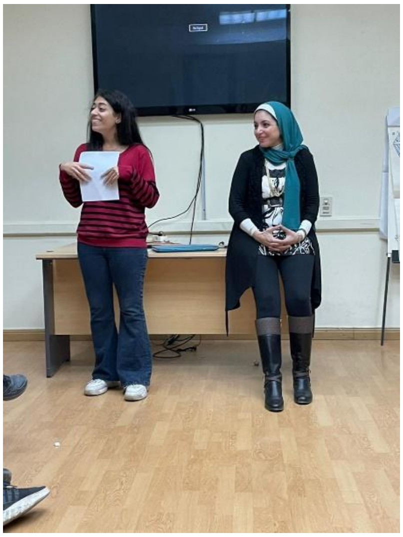

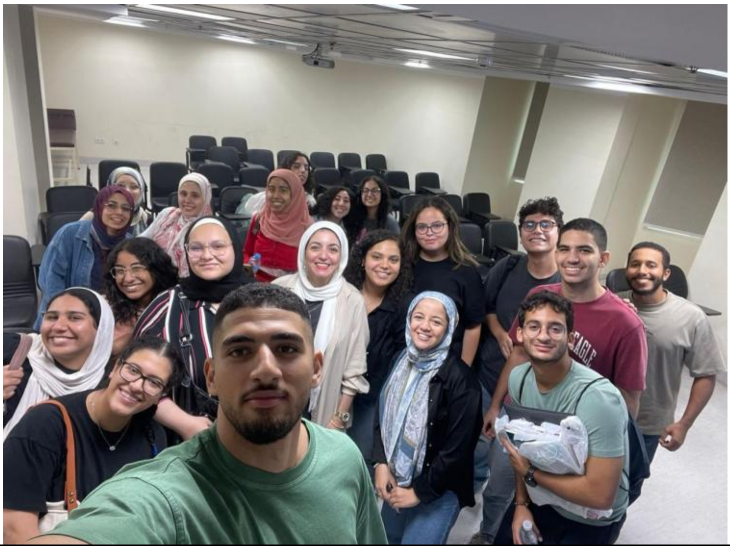

the origami 3D heart activity

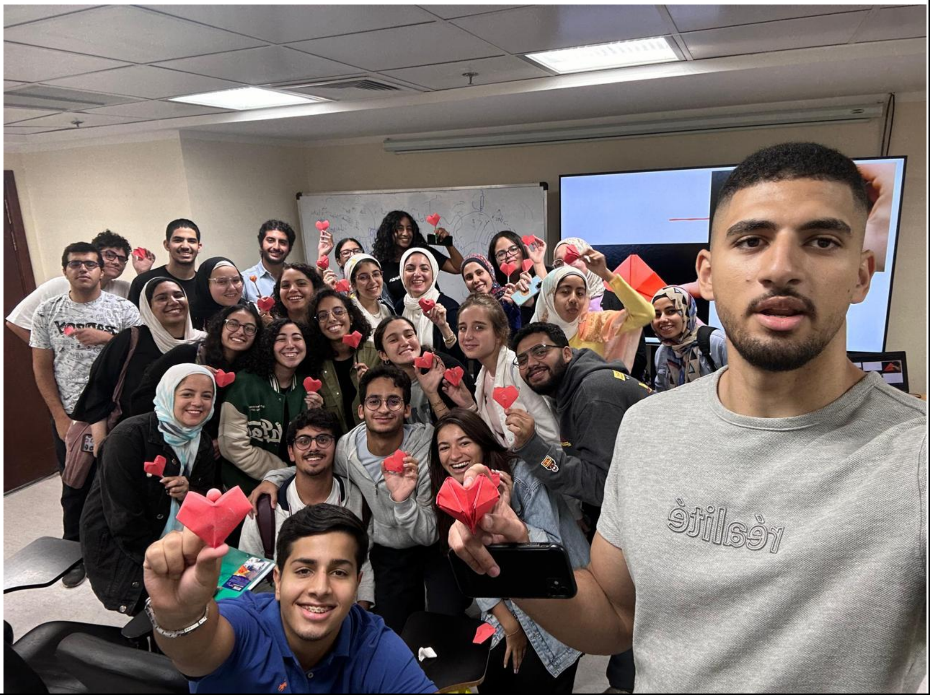

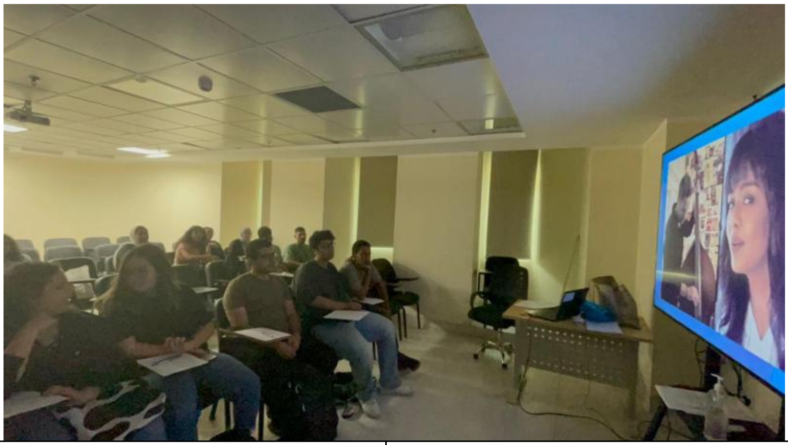

The hope notes activity

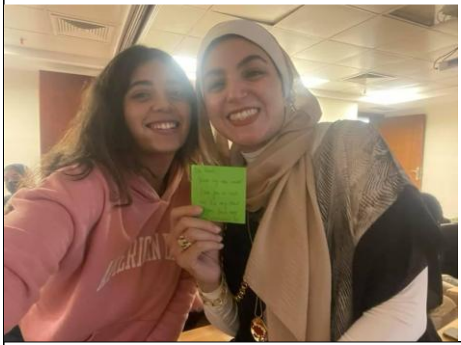

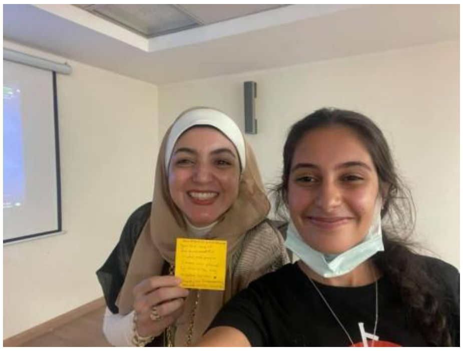

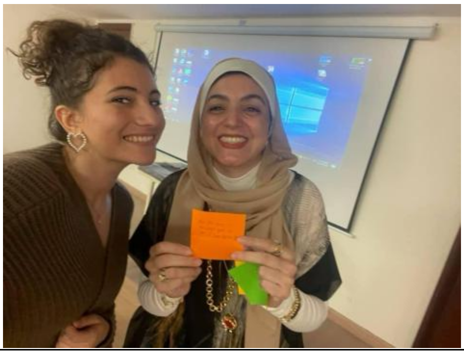

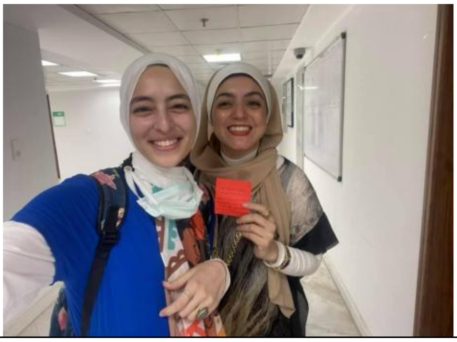

Appendix (7)

Reserch ethics committee approval letter-AFCM

رقم التعديل<br>(_)	رقم الإصدار<br>(01)	كود الوثيقة<br>PGSR-SREC-02-02-04-11	لجنة اخلاقيات البحث العلمي	كلية الطب بالقوات المسلحة
تاريخ التعديل<br>/ /	تاريخ الاصدار<br>8/2023	Page 41 of 41	الموافقة على البحث للانسان	

Approval form

Date: 25 / 11 /2023
Serial number of the protocol: 383
Name of the Principle investigator: Rania Moustafa Kamel Abdelbaset Moustafa Hadhoud
Title: Evaluating the Impact of an Emotional Intelligence Elective Course: A Quasi-Experimental Study in an Egyptian Medical School

This is to certify that the institutional review board At Armed Forces College of Medicine, Cairo, Egypt (65 meeting 25-11-2023) has approved your research and did not find any ethical violations. It is the responsibility of the principal investigator to safeguard the rights and welfare of human subjects involved in the research. An ethical approval is an overall strategy that describes how the rights and welfare of human subjects who participate in research studies are safeguarded.

Any changes to the design or methodology of the approved research should be reported to the institutional review board for the approval before implementation.
Principle investigator should provide us with a six monthly progress report.

Reviewed by:

Brig. Dr. Nashwa Ibrahim **L. Col. Mohamed Fekry** **Mr. Ahmed Mamdouh**

IRB Vice Chair
Maj. Gen. Med. Hany Hafez

IRB Chair
Maj. Gen. Med. Eid El Tawil

AFCM - IRB
Armed Forces College of Medicine

Appendix (8)
Research approval letter from the Head of the Extended Modula Program (EMP)-ASU

EMP
Extended Modular Program

Faculty of Medicine-Ain Shams University

Request for Administrative Approval to Proceed with Thesis Requirements

Dear Prof, Director of the Extended Modular Program (EMP)-ASU

I hope this letter finds you well. I, Ass, Prof. Rania Mustafa Kamel Abdel Basset Hadhoud, Assistant Professor of Forensic Medicine and Toxicology at Ain Shams University, have successfully completed the initial phase of my program and am now preparing to fulfill the remaining thesis requirements. I am writing to formally request your administrative approval to proceed with the necessary documentation required by the Ethics Committee at the Faculty of Medicine, Armed Forces, for my master's degree in Medical Education.

Thesis Title:

Evaluating the Impact of an Emotional Intelligence Elective Course on Third-Year Medical Students Using the Kirkpatrick Model: A Quasi-Experimental Study in an Egyptian Medical School

This research aims to evaluate the effectiveness of the Emotional Intelligence elective course, which I instruct for third-year students in the Expanded Medical Program (EMP). The study will assess:

1. Student satisfaction (Kirkpatrick Level 1 - Reaction) through post-course evaluations.

2. Knowledge and skill acquisition (Kirkpatrick Level 2 - Learning) via pre- and post-test comparisons.

Methodology:

1. Course Evaluation Survey: A 5-point Likert scale questionnaire to gauge participant satisfaction.
2. Pre- and Post-Assessment: A 22- question instrument aligned with course learning outcomes to measure knowledge, skills, and values.
3. Emotional Intelligence Measurement: The Self-Report Emotional Intelligence Test (SSEIT) to evaluate three domains: emotional perception, regulation, and utilization.

I have attached the full research protocol for your review. Your approval will enable me to advance this study, which contributes to the growing body of knowledge on emotional intelligence in medical education.

Thank you for considering this request. I greatly appreciate your support and am available to address any questions or provide additional information as needed.

Sincerely,

Dr. Rania Mustafa Hadhoud

Assistant Professor of Forensic Medicine and Toxicology

Ain Shams University

approved

Ain Shams Faculty Of Medicine
EMP
Extended Modular Program

Appendix (9)

Reserch ethics committee approval letter-ASU

Community, Environmental and Occupational Medicine Department

Ain Shams University

Methodological Approach of a Research Protocol

A Review Checklist

Title of the Research: Evaluating the Impact of an Emotional Intelligence Elective Course: A Quasi-Experimental Study in an Egyptian Medical School
Name of Researcher: Rania Moustafa Kamel Abdelbaset Hadhoud
Specialty: Forensic Medicine and Toxicology
Type of Research: Post-doctorate Paper

I- Research Objective

   a. Well formulated objective: √

Proposed reformulated objective: To compare the changes in students' emotional intelligence-related knowledge and reported skills before and after an elective course at Ain Shams University.

Objective conforming to study design √

II- Study Design Review

   a. Type of Study Design: -----

   b. Proposed study design: Quasi-Experimental Study (Pre/Post design)

III- Sample Size:

Using G power program for sample size calculation: setting power at 90% and α error at 5%, a sample size of 32 third year medical students at Ain Shams University can detect a statistically significant difference between before and after the emotional intelligence (EI) elective course as regard knowledge score toward emotional intelligence measured by The Schutte Self Report Emotional Intelligence Test (SSEIT), assuming a medium to large effect size difference (dz=0.6) regarding ***Kloth et al., 2022***, using two-sided t-test (difference between two dependent means).

Assuming that dropout is of 10%, a sample size of **at least 36 third year medical students receiving EI elective course at Ain Shams University** will be needed.

IV- Missed Items to be added: ---------------

Name of Reviewer: Dr. Shaimaa Samy Yousef **Date:** 8/6/2024

CEOM

Presented for Medical Research Ethical Committee at Faculty of Medicine Ain Shams University

Review Checklist

APPENDIX (10)

Research support unit sample size calculation letter

Armed Forces College of Medicine (AFCM)

Research Support Unit (RSU)

Sample Size Calculation for Research Proposal

Title of the research *	Evaluating the Impact of an Emotional Intelligence Elective Course: A Quasi-experimental Study in an Egyptian Medical School
Date sent (dd/mm/yy)	13/09/2023
Principle investigator *	Prof. Rania Moustafa Kamel Abdel-Baset Hadhoud
Department *	Medical Education Development Unit
Phone number *	01223747656
E-mail *	raniamhadhoud@gmail.com / hadhoud.mee801batch2@gmail.com
Study design *	A quasi-experimental study, involving a pretest and a posttest. [O1 - X - O2]
Study objectives *	1. To assess third-year medical students' satisfaction with the emotional intelligence elective course at the first level Kirkpatrick evaluation model (Reaction) in Ain Shams University<br>2. To evaluate the changes in students' emotional intelligence-related knowledge and reported skills after completing the course at the second level of the Kirkpatrick evaluation model (Learning) in Ain Shams University
Primary outcome *	The impact of emotional intelligence elective course on third-year medical students at the extended modular program -Faculty of Medicine -Ain Shams University according to satisfaction and acquisition of emotional intelligence-related knowledge and skills
Parameters for sample size calculation **	Students' performance
Software used for calculation **	Data will be revised for completeness, accuracy, and logical consistency. Pre-coded data will be entered on the computer using the statistical package of social science software program, version 21 (SPSS) to be statistically analyzed. Data will be summarized using mean, SD, median and IQR for quantitative variables and number and percent for qualitative variables. comparison between qualitative variables will be done using chi-square test for qualitative variables while independent test used to compare quantitative variable between two groups, one way ANOVA test for quantitative variable between more than two categories which where normally distributed and nonparametric Kruskal -Wallis and Mann-Whitney tests for quantitative variables which were not normally distributed. Other statistical tests used

	when appropriate. P value less than 0.05 was considered of statistical significance
Reference (s) article used for calculation **	Edussuriya, Deepthi & Perera, Sriyani & Marambe, Kosala & Wijesiriwardena, Yomal & Ekanayake, Kasun. (2022):The associates of Emotional Intelligence in medical students: A systematic review. The Asia Pacific Scholar. 7. 10.29060/TAPS.2022-7-4/OA2714.<br>Kloth C, Schmidt SA, Graeter T, Nikolaou K, Kaufmann S, Beer M, Thaiss WM.(2022): Evaluation of an elective ultrasound course for medical students. Clin Anat. 2022 Apr;35(3):354-358. doi: 10.1002/ca.23838. Epub Feb 8. PMID: 35128729.<br>Heydari, M.R., Taghva, F., Amini, M. et al. (2019):Using Kirkpatrick's model to measure the effect of a new teaching and learning methods workshop for health care staff. BMC Res Notes 12, 388 . https://doi.org/10.1186/s13104-019-4421-y
Minimum required sample size (statement to be written in the research methodology section) **	A sample size of 36 students attending the course out of the total population sample (120 students) would be sufficient to achieve the study objectives
Date completed **	28/10/2023
Sample size calculation done by **	Dr Hanaa Zaghloul Yousof

*: To be filled by the applicant
**: Left blank (filled by the research support unit)
